# Forced association of SARS-CoV-2 proteins with the yeast proteome perturb vesicle trafficking

**DOI:** 10.15698/mic2021.12.766

**Published:** 2021-10-27

**Authors:** Cinzia Klemm, Henry Wood, Grace Heredge Thomas, Guðjón Ólafsson, Mara Teixeira Torres, Peter H. Thorpe

**Affiliations:** 1 School of Biological and Behavioural Sciences, Queen Mary University of London, E1 4NS, UK.; 2 Institute for Systems Genetics, NYU Langone Health, New York, NY 10016, USA.

**Keywords:** SARS-CoV-2, COVID-19, synthetic physical interactions, vesicle trafficking, transcription

## Abstract

Severe acute respiratory syndrome coronavirus 2 (SARS-CoV-2) is the causative agent of the highly infectious coronavirus disease COVID-19. Extensive research has been performed in recent months to better understand how SARS-CoV-2 infects and manipulates its host to identify potential drug targets and support patient recovery from COVID-19. However, the function of many SARS-CoV-2 proteins remains uncharacterised. Here we used the Synthetic Physical Interactions (SPI) method to recruit SARS-CoV-2 proteins to most of the budding yeast proteome to identify conserved pathways which are affected by SARS-CoV-2 proteins. The set of yeast proteins that result in growth defects when associated with the viral proteins have homologous functions that overlap those identified in studies performed in mammalian cells. Specifically, we were able to show that recruiting the SARS-CoV-2 NSP1 protein to HOPS, a vesicle-docking complex, is sufficient to perturb membrane trafficking in yeast consistent with the hijacking of the endoplasmic-reticulum–Golgi intermediate compartment trafficking pathway during viral infection of mammalian cells. These data demonstrate that the yeast SPI method is a rapid way to identify potential functions of ectopic viral proteins.

## INTRODUCTION

SARS-CoV-2 is a novel member of the *Coronaviridae* family of single-strand RNA viruses. Both the current SARS-CoV-2 pandemic and two previous instances of human infections with novel coronaviruses (SARS-CoV and MERS-CoV), demonstrate the potential of this virus to cause widespread infections and associated mortality. Infection may cause acute respiratory distress syndrome characterised by severe inflammation of the lungs, which can lead to short term mortality and long-term lung damage. Despite the number of antiviral drugs being tested such as remdesivir, hydroxychloroquine, lopinavir and interferon, to date, all of these agents had little or no effect on rates of mortality or duration of hospitalisation [1]. Although vaccines offer up to 90% protection against catching the disease, the treatment of acute infection relies upon a small number of anti-inflammatory drugs, which target the patient's immune response rather than act against the viral infection [2]. Therefore, there is much interest in understanding the function of coronavirus proteins, both in terms of the host proteins with which they interact and the cellular processes that they exploit during viral infection. However, with notable exceptions, the functions of many of the SARS-CoV-2 proteins are either unknown or poorly characterised.

Large scale proteomic analysis using mass spectrometry has already enabled significant insight into the molecular mechanisms of specific SARS-CoV-2 proteins during coronavirus infection. Immuno-precipitation (IP) experiments with SARS-CoV-2 proteins in human cells have identified interactions between viral and host proteins and have proven to be a powerful tool to identify drug targets within the human cell [4, 5]. However, some interactions may remain undetected due to the technical limitations of mass spectrometry.

Testing the function of individual SARS-CoV-2 proteins systematically is appealing to determine their potential role in cells and ultimately to develop interventions to block their activity. Notwithstanding the IP experiments, there are relatively few methods for querying the function of proteins in an unbiased and systematic way. We wished to determine whether individual SARS-CoV-2 proteins would produce a phenotype when expressed in yeast and to identify whether such a simple system could provide an assay for viral protein function. The rationale for using a simple eukaryote to study viral protein function is to ask whether yeast proteins are sufficiently conserved to be affected by the SARS-CoV-2 proteins and potentially also provide an *in vivo* assay for their function. If the viral proteins exclusively interact with mammalian-specific proteins or processes, they are unlikely to produce a phenotype in a non-mammalian model. In contrast, if viral proteins exploit conserved pathways, they may be capable of eliciting a response in yeast. There is significant precedent for studies using ectopic proteins in budding yeast, from studying diseases such as Huntington's disease or cancer. However, relatively little evidence had been gained on potential assays for viral proteins in yeast. To this end, we undertook Synthetic Physical Interactions (SPI) screens with seven SARS-CoV-2 proteins in yeast. In brief, SPI screens take a protein of interest, a viral protein in this case, and sequentially force it to associate with most members of the yeast proteome. Forced associations that result in a growth defect are readily detected and we term these associations SPIs. Interactions that affect the cell cycle or essential cellular functions, when forced constitutively, produce a yeast growth defect. Consequently, SPI screens have been used to uncover novel functional roles for proteins in budding yeast [6–8].

## RESULTS

### SARS-CoV-2 proteins localise to the vacuole and components of the ER-Golgi intermediate compartment trafficking pathway in yeast

**Figure 1:**
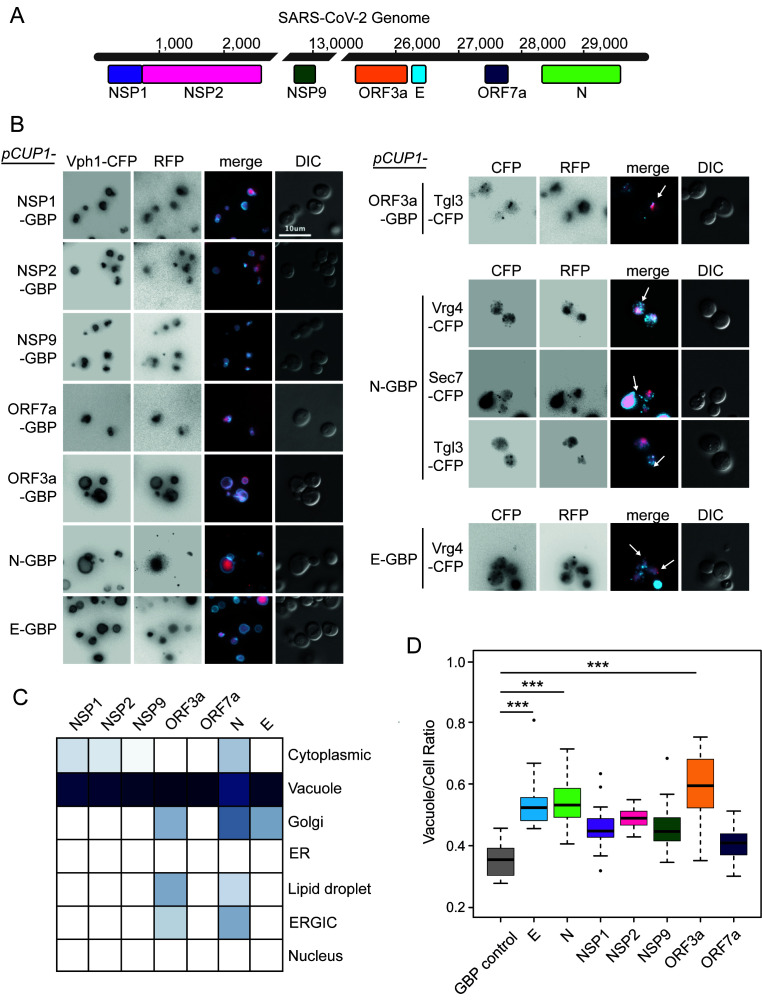
FIGURE 1: Localisation of SARS-CoV-2 proteins in yeast. **(A)** Seven open reading frames (ORFs) from the SARS-CoV-2 viral genome are illustrated. These ORFs were cloned either in fusion with GBP-GFP or alone and transferred to the GFP collection of strains and growth effects measured. The viral ORF alone and GBP-RFP serve as controls. The respective colours of the seven viral proteins are consistent in data across the remaining figures. **(B)** Fluorescence micrographs show yeast encoding different CFP-tagged yeast proteins to evaluate cellular position of the GBP-RFP-tagged SARS-CoV-2 proteins. Vph1-CFP is a marker for the vacuolar membrane, Tgl3-CFP for lipid droplets, Vgr4-CFP for the Early Golgi, and Sec7-CFP for the late Golgi. The blue and red channels are shown in greyscale for clarity. The scale bar is 10 µm, note that GBP does not bind to CFP. **(C)** A summary of SARS-CoV-2 protein localisation based on CFP-marker co-localisation. The blue colour is a representation of the percentage of cells with the SARS-CoV-2 protein co-localised with a specific compartment (dark blue=100%, white=0%; n=~50 cells per strain). **(D)** Yeast expressing the SARS-CoV-2 proteins have a larger vacuole/cell ratio. The boxplots show median ratio (dark bar) and box extends from the lower (0.25) to upper (0.75) quartiles. The error bars show the minimum and maximum values, defined as either the actual minimum and maximum or 1.5 times the inter-quartile range below and above the lower and upper quartiles respectively. Outliers are shown as dots. The triple asterisks, *** indicate a p value <0.001 using an ANOVA test, n=15 cells per strain. The control strain contains GBP-RFP with no viral protein.

We initially tested whether or not the expression of SARS-CoV-2 proteins in yeast would result in a growth defect. The 30 kb genome of SARS-CoV-2 contains up to 14 open reading frames (ORFs), which encode for at least 29 proteins. These include the key structural proteins spike (S), envelope (E), membrane (M) and nucleocapsid (N) as well as 16 non-structural proteins (NSP1-16) and several accessory proteins (known as ORFs 3a, 3b, 6, 7a, 7b, 8, 9b, 9c and 10) (**Figure 1**). We chose seven proteins encompassing members of each of these groups; E, N, NSPs 1, 2 and 9 and ORFs 3a and 7a to investigate in yeast. E forms a homopentameric ion channel, and much of the expressed protein localises to the ER, Golgi and ER-Golgi intermediate compartment (ERGIC), where it participates in virion assembly and budding [9, 10]. It has been shown to interact with M, N, and five human host proteins Bcl-xL, PALS1, Syntenin, ATP1A1, and Stomatin [11]. ORF3a is a homotetrameric ion channel that has been suggested to modify endomembrane compartments and interacts with the vacuolar protein sorting complex, HOPS [4]. It may also play a role in viral release and host cell apoptosis [12, 13]. N primarily functions to bind to viral RNA, and is essential to package the RNA into the nascent virions [14]. NSP1 inhibits the expression of host proteins and affects nuclear pores [15]. NSP2 is a transmembrane protein and interacts with vesicle trafficking proteins [4]. NSP9 is involved in the translation of viral RNAs and interacts with the translation initiation factor eIF4A and components of the nuclear pore [4]. Overexpression of ORF7a has been shown to induce Golgi fragmentation caused by SARS-CoV-2 infection [16]. These studies have identified some putative roles of the viral proteins, but little is known about the mechanisms that they work through.

Plasmid constructs were created encoding these seven SARS-CoV-2 proteins (**Figure 1A**) under the control of a modestly, but constitutively active *CUP1* promoter. We observed no obvious growth defects in strains expressing any one of the seven viral proteins, whether or not they were tagged with green fluorescent protein (GFP)-binding protein-red fluorescent protein (GBP-RFP; Figure S1). Next, we wanted to identify the localisation of the viral proteins within yeast cells. We used plasmids encoding fluorescently-tagged versions of each of the SARS-CoV-2 proteins (tagged with GBP-RFP) and transfected these into a set of yeast strains each of which contained a cyan fluorescent protein (CFP)-tagged endogenous protein that localises to a specific yeast compartment (Figure S2); note that the GBP does not interact with CFP. We found that all SARS-CoV-2 proteins are predominantly targeted to the vacuole, a known pathway for protein degradation of recombinant and misfolded proteins [17–19] (**Figure 1B, C** and Data S1). In addition, ORF3a, N and E partially colocalised to components of the ER-to-Golgi pathway and Lipid Droplets (**Figure 1C**). Interestingly, expression of these SARS-CoV-2 proteins also leads to an increase in the ratio of vacuole to cell size which is tuned by anterograde membrane trafficking [20] (**Figure 1D** and Data S1).

### Proteins involved in cellular trafficking and RNA metabolism are sensitive to forced SARS-CoV-2 protein association

**Figure 2:**
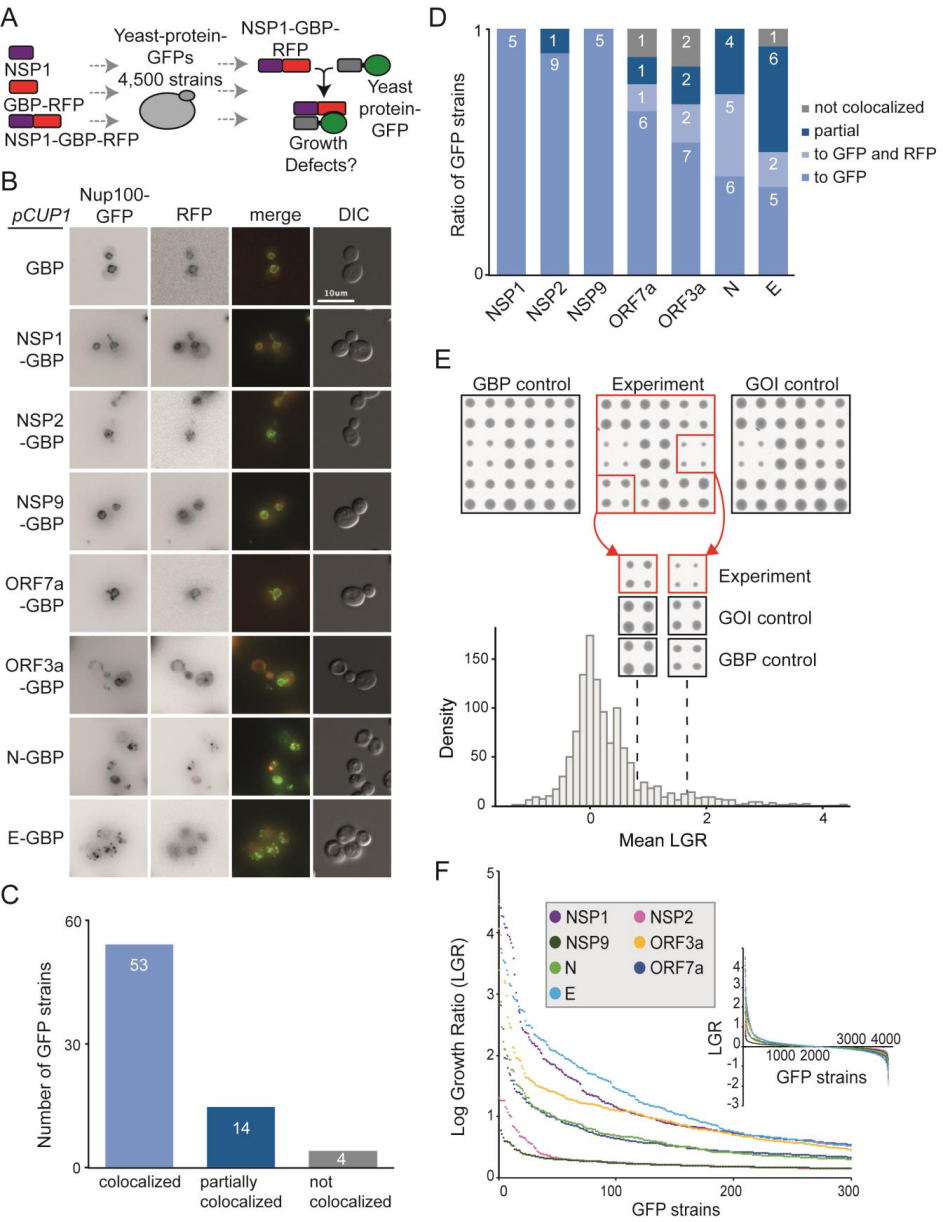
FIGURE 2: Forced binding of SARS-CoV-2-GBP-RFP fusion proteins to the yeast proteome. **(A)** The schematic illustrates the SPI screen, which involved recruiting SARS-CoV-2 proteins (tagged with GBP) to different GFP proteins, the viral protein alone and GBP alone serve as controls. **(B)** Fluorescence micrographs of a Nup100-GFP strain containing different viral proteins fused with GBP-RFP are shown. The green and red channels are shown in greyscale for clarity, the scale bar is 10 µM. **(C)** 71 strains were selected that contain different SARS-CoV-2 proteins tagged with GBP-RFP and different GFP tagged proteins. The GFP tagged proteins were chosen based upon those that had a clearly-visible GFP signal that is located at a defined and easily recognisable location within the cell. Co-localisation of GFP to RFP signal was assessed using fluorescence imaging. 67 of these strains (94%) had GFP and RFP co-localised or partially co-localised. None of the imaged GFP strains were completely recruited away from their natural cellular localisation. **(D)** The colocalisation data shown collectively in panel C, are separated by each viral protein. In some cases, in N, E ORF7a and ORF3a SPIs, the fusion protein localises to both to the normal location of the GFP protein as well as to the SARS-CoV-2 location described in Figure 1 (e.g. vacuole). This is labeled as ‘localises to GFP and RFP'. **(E)** Sample images from the screen are shown (top), GFP strains are arrayed in quadruplicate. Colony size is used as a measure of growth. The natural logarithm of the average growth of the control divided by the experiment (log growth ratio, LGR) are calculated. High LGR values indicate that control colonies were larger than the experiment. GOI indicates ‘Gene Of Interest'. The dotted lines indicate the mean LGR value for the examples shown. **(F)** LGRs between yeast proteome and seven SARS-CoV-2-GBP-RFP fusion proteins are shown (inset) and the strongest 300 SPIs with the highest LGR from all screens are plotted.

Next, we wanted to ask whether forcibly associating viral proteins with endogenous yeast proteins *in vivo* would lead to growth defects. We first tested whether the SARS-CoV-2 proteins, when fused to GBP, were able to associate with endogenous yeast proteins. We imaged a selection of yeast GFP strains expressing SARS-CoV-2 proteins fused with GBP. We found that, in the majority of cases, the SARS-CoV-2 proteins tagged with GBP co-localised with yeast GFP proteins as judged by fluorescence imaging (**Figure 2A-D** and Figure S3), consistent with previous findings for yeast proteins [6]. We next performed SPI screens with the seven SARS-CoV-2 proteins. We arrayed ~4000 strains from the GFP library of yeast strains on rectangular agar plates. This subset of the full GFP library excludes tagged proteins that are either non-functional or not expressed in mitotic cells [21]. Each strain was assessed in quadruplicate. We used Selective Ploidy Ablation (SPA) [22] to transfer plasmids encoding the seven GBP-tagged SARS-CoV-2 proteins into the arrays of GFP strains. As controls, we separately screened untagged SARS-CoV-2 proteins and the GBP protein alone. Growth of the GFP strains was compared between these two controls and the strains containing a SARS-CoV-2 protein tagged with GBP. Colony size was used as a surrogate for growth and the natural log of the ratio of growth (control growth divided by experimental growth) was calculated for each of the ~28,000 associations. A Log Growth Ratio (LGR) of zero indicates equivalent growth on control and experiment, whereas positive LGRs indicate restricted growth of the experimental strains (**Figure 2E**, Data S2). The LGRs for each of the two controls were compared (Figure S4) and since the data for each control correlated, we used a ‘mean LGR' of the two controls as a quantitative measure of any SPIs, consistent with previous SPI screens [6–7]. We categorised a SPI as a forced association that produces a mean LGR of 0.4 or greater; this corresponds to roughly twice as many cells on control versus experiment [8]. Previous SPI screens have shown that a mean LGR of 0.4 is a conservative cut-off, with an estimated false discovery rate (FDR) of around 90% [6, 7]. To measure the FDRs directly we validated a selection of the SARS-CoV-2 SPIs by repeating the assay with 16 replicates. We found that the original screen data correlated well with the validation data (Figure S5A-D). Overall, the original SARS-CoV-2 SPIs had an FDR of up to 20% with a conservative 0.4 LGR cut-off. SPIs with higher original LGR values validated at higher levels (Figure S5F-H).

We removed yeast proteins from this dataset that frequently are affected by protein association using the SPI method, so-called ‘frequent flyers' [6] (Data S2). Two of the SARS-CoV-2 proteins, NSP2 and NSP9 produced very few SPIs (32 and 17 respectively). Two others, ORF7a and N, produced around 200 SPIs (215 and 201 respectively) and the remainder produced 300-500 SPIs (335 for ORF3a, 394 for E and 432 for NSP1 (**Figure 2E, F** and Data S2).

**Figure 3:**
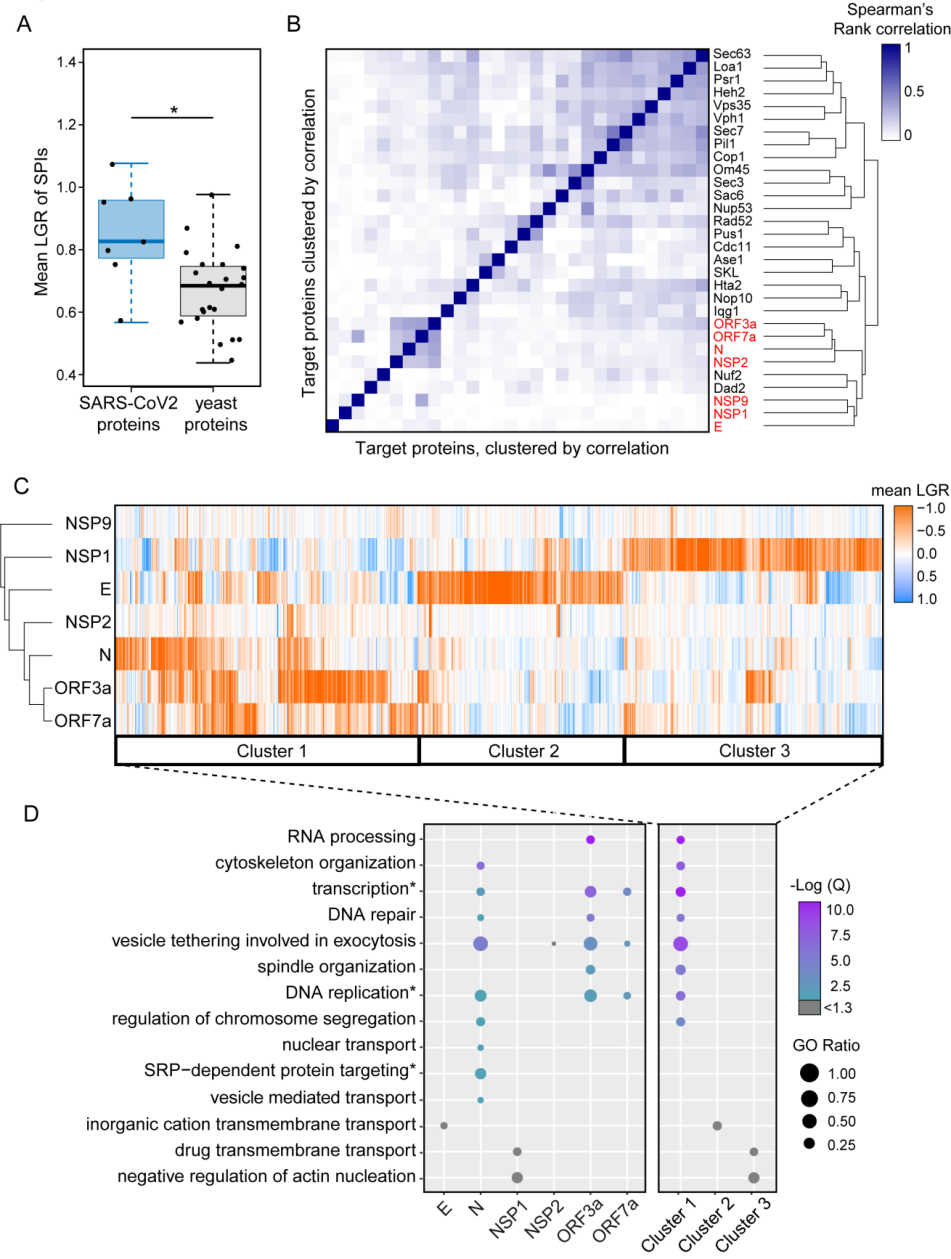
FIGURE 3: Clustering of SPIs from SARS-CoV-2 screens and yeast screens. **(A)** Compared to previous SPI screens which use yeast proteins of interest, the SARS-CoV-2 SPIs, on average, have higher LGRs. Statistical significance was assessed using Welch's two sample t-test (the two asterisks, * indicate a p-value of = 0.029). Boxplot parameters are as in Figure 1D. **(B)** Hierarchical clustering of Spearman's rank correlation between the LGRs from all screens indicate that SARS-CoV-2 screens (red) are less correlated to yeast screens, except for Nuf2 and Dad3. The darker blue colours indicate stronger correlation of LGR data. **(C)** Proteins identified as SPIs in one of more screens out of the seven viral protein screens were clustered based upon their mean LGR values using centroid linkage. Three distinct clusters of GFP proteins were identified (Cluster 1-3). **(D)** Enrichment analysis of gene ontology (GO) biological process terms across the seven screens and clusters from panel C. P values were calculated using a hypergeometric test and corrected for multiple hypothesis testing to produce a false discovery rated corrected, Q-value. The negative logarithm of the Q value is shown from purple (high) to turquoise (low); a -Log(Q) value of 1.3 is equivalent to p=0.05, with higher -Log(Q) values indicating greater statistical significance. -Log(Q) values below 1.3 are shown in grey. GO ratio (size of the circle) indicates the proportion of genes in a GO term were found for each enrichment.

We noted that the SARS-CoV-2 SPIs produced particularly strong growth inhibition compared to those using yeast proteins. To confirm this, we measured the mean LGRs of SPIs produced by the seven proteins and compared this with 23 yeast proteins representing diverse cellular compartments [6]. We found that, on average, the SARS-CoV-2 proteins produced stronger growth effects than yeast proteins (p=0.029, **Figure 3A**). This was particularly true for the SARS-CoV-2 proteins that produced the most SPIs. We next used Spearman's rank correlation to identify similarities between yeast and SARS-CoV-2 SPI screens. We found that the SARS-CoV-2 SPI data showed poor correlation to the 23 yeast proteins with two exceptions, Nuf2 and Dad2, which are both kinetochore proteins (**Figure 3B**). The NSP1 and E screens both produced SPIs with distinct sets of GFP-tagged proteins, whereas screening with N, ORF3a and ORF7a resulted in similar sets of SPIs (**Figure 3B**).

We used hierarchical clustering on the SPI data to group the plasmid-expressed SARS-CoV-2 proteins and the GFP-tagged yeast collection by LGRs (**Figure 3C**). This analysis shows that the most closely related SPI data are for ORF3a and ORF7a, with the N protein also having some overlap with these data. The E and NSP1 proteins separately produced large and distinct groups of SPIs.

Gene ontology enrichment (GO) analysis was used to assess enrichment of specific cellular processes in SPIs from each screen individually (**Figure 3D**, Data S3). Enrichments are defined as GO terms with an FDR corrected q-value ≤ 0.05 (-Log(Q) ≥ 1.3) and GO terms above this q-value threshold are shown in grey. NSP9 produced very few SPIs and therefore had no enrichment. NSP2 SPIs included proteins involved in vesicle tethering and vesicle mediated transport although these GO terms were not enriched after FDR correction (Data S3). ORF3a, ORF7a and N SPIs shared similar GO enrichments, including vesicle tethering involved in exocytosis, transcription, DNA repair and replication. The GO terms SRP-dependent protein targeting, nuclear transport and regulation of chromosome segregation were uniquely enriched in SPIs with N. OFR3a SPIs were additionally enriched for proteins which play a role in RNA processing and spindle organisation (**Figure 3D** and Data S3). Despite being above the FDR corrected q-value threshold, the ion channel E produced SPIs with proteins involved in inorganic cation transmembrane transport, which underline its proposed function as an ion channel. NSP1 SPIs included proteins involved in transmembrane drug transport, regulation of vesicle transport and regulation of actin nucleation (**Figure 3D** and Data S3). Rather than producing a uniform ‘random' list of proteins, our analysis indicates that the SARS-CoV-2 SPIs are enriched with proteins involved in distinct biological processes.

**Figure 4:**
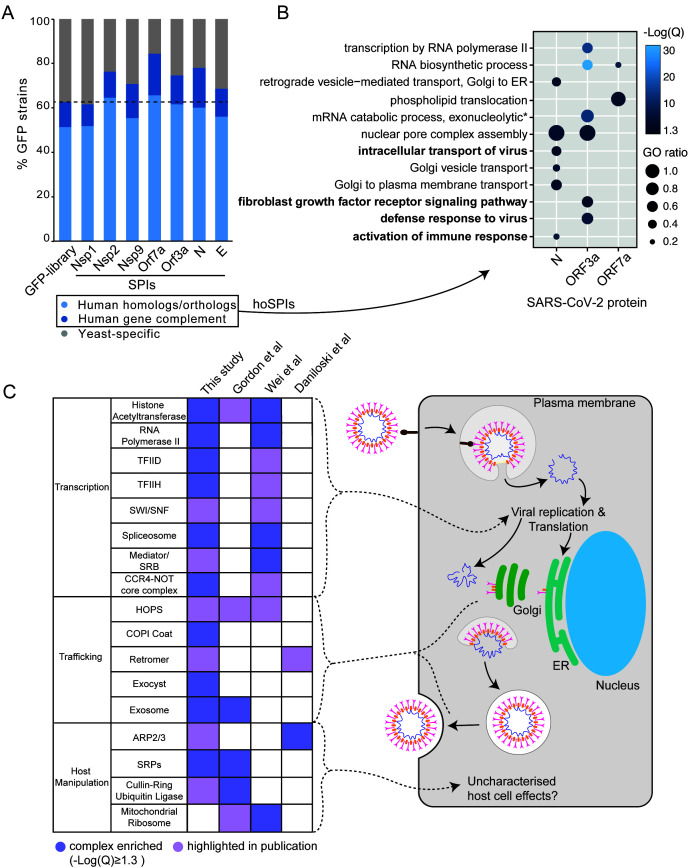
FIGURE 4: SARS-CoV-2 SPI screens in the context of human genetics. **(A)** The SARS-CoV-2 yeast SPIs are collectively enriched for those that have human homologues or orthologues compared to the GFP strains tested (Fishers exact test, p=7.8x10^-8^). Human Gene Complement indicates those human genes that have been demonstrated to complement the loss of the yeast homolog/ortholog. **(B)** Enrichment analysis of GO biological process terms of human orthologues of the SARS-CoV-2 SPIs (hoSPIs). Bold terms indicate human specific GO terms, that are not present in yeast. The -Log(Q) value is illustrated by blue shading, all -Log(Q) values are greater than or equal to 1.3, which is equivalent to q-values < 0.05. GO ratio (size of the circle) indicates the proportion of genes in a GO term were found for each enrichment. **(C)** A comparison of the complexes that contain hoSPIs with those identified from three SARS-CoV-2 screens performed in mammalian cells. Dark purple shading indicates enrichment, light purple shading highlights complexes which are mentioned by the authors. These complexes correspond to the various steps in the viral lifecycle (right).

In order to determine whether the yeast SPI system could provide functional insight into the SARS-CoV-2 proteins, we focused on SPIs with proteins that are conserved from yeast to human cells. More than 60% of GFP-tagged proteins in our yeast library have known orthologues or homologues in human cells (**Figure 4A**). Using Yeastmine's orthology tool [23], we identified 1137 human orthologues of the SPIs (henceforth referred to as hoSPIs) (Data S4). With GO enrichment analysis, we asked whether hoSPIs were enriched for specific pathways. We found that, as with the yeast enrichments (**Figure 3D**), the hoSPIs were enriched for some similar functional categories (**Figure 4B**, Data S4). For example, the hoSPIs of N were enriched for proteins involved in vesicle transport (e.g., Golgi vesicle transport or vesicle mediated transport, Data S4). Additionally, the N hoSPIs were enriched for mammalian specific processes such as the intracellular transport of virus and activation of the immune response (Data S4). Also, similar to the yeast enrichments, ORF3a hoSPIs were enriched for processes involving RNA synthesis (e.g., RNA biosynthetic processes or regulation of gene expression, Data S4). Furthermore, ORF3a hoSPIs were enriched for human-specific processes such as defense to virus (Data S4). These data show that the hoSPIs were enriched for proteins that are involved in RNA metabolism, vesicle transport and other aspects of the immune response or response to viruses.

We next compared the hoSPIs to recent studies which identified cellular targets of SARS-CoV-2 infection in mammalian cells. Firstly, we compared our hoSPIs to affinity-purification mass spectrometry (AP-MS) data of SARS-CoV-2 proteins expressed in HEK-293T/17 cells (Gordon *et al.* [4]). We only found four hoSPIs from the N screen that overlapped with proteins that co-purified with N in human cells detected by AP-MS), these were MRPL36, G3BP1/2, LARP1B and PABPC1/2/RBM28. HoSPIs from other screens did not overlap with their corresponding AP-MS screens from the Gordon *et al.* data. However, we identified a total of 18 other hoSPI proteins that were detected with different SARS-CoV-2 proteins in the AP-MS data (Figure S6, Data S5). Since forced protein association can affect neighbouring proteins, depending on the specific spatial arrangement of the GBP- and GFP-tagged proteins [24], we also looked for correlation between protein complexes that were identified in the different screens. The proteins identified by AP-MS were enriched for proteins that are part of the signal recognition particle (SRP) and the exosome (**Figure 4C** Data S5). This same GO term was enriched in the hoSPIs (Data S5). Additionally, the AP-MS study highlighted histone acetylase complex, cullin-RING ubiquitin ligase complex and the HOPS complex, components of which were identified within the hoSPIs (**Figure 4C**, Data S5). We also compared protein complexes containing hoSPIs to data from two recent CRISPR knockout screens in SARS-CoV-2-infected mammalian cells. First, we examined genes that impacted lethality to SARS-CoV-2 (with Z score of >+1, <−1) in monkey VeroE6 cells identified in a screen by Wei and colleagues [24] (Data S5). We found that similar GO terms were enriched between the protein complexes containing hoSPIs and complexes identified from this CRISPR knockout screen. These included various protein complexes related to transcription, histone acetyltransferases and the spliceosome (**Figure 4C** and Data S5). Additionally, we noted that complexes, highlighted by Wei and colleagues, also contained hoSPI proteins (**Figure 4C**). We next compared the hoSPIs with the 1024 genes identified by Daniloski and colleagues in another CRISPR SARS-CoV-2 loss-of function screen [25]. We found overlapping proteins within the retromer and ARP2/3 complexes (**Figure 4C**), although these were not enriched for GO terms in our SPI data. In summary, we identified hoSPIs within mammalian protein complexes that were shown to either interact with viral proteins [4] or are essential for viral function and replication [25, 26]. These data establish that the yeast SPI system can identify interacting pathways with the SARS-CoV-2 viral proteins in eukaryotic cells despite the extensive divergence of yeast and humans.

**Figure 5:**
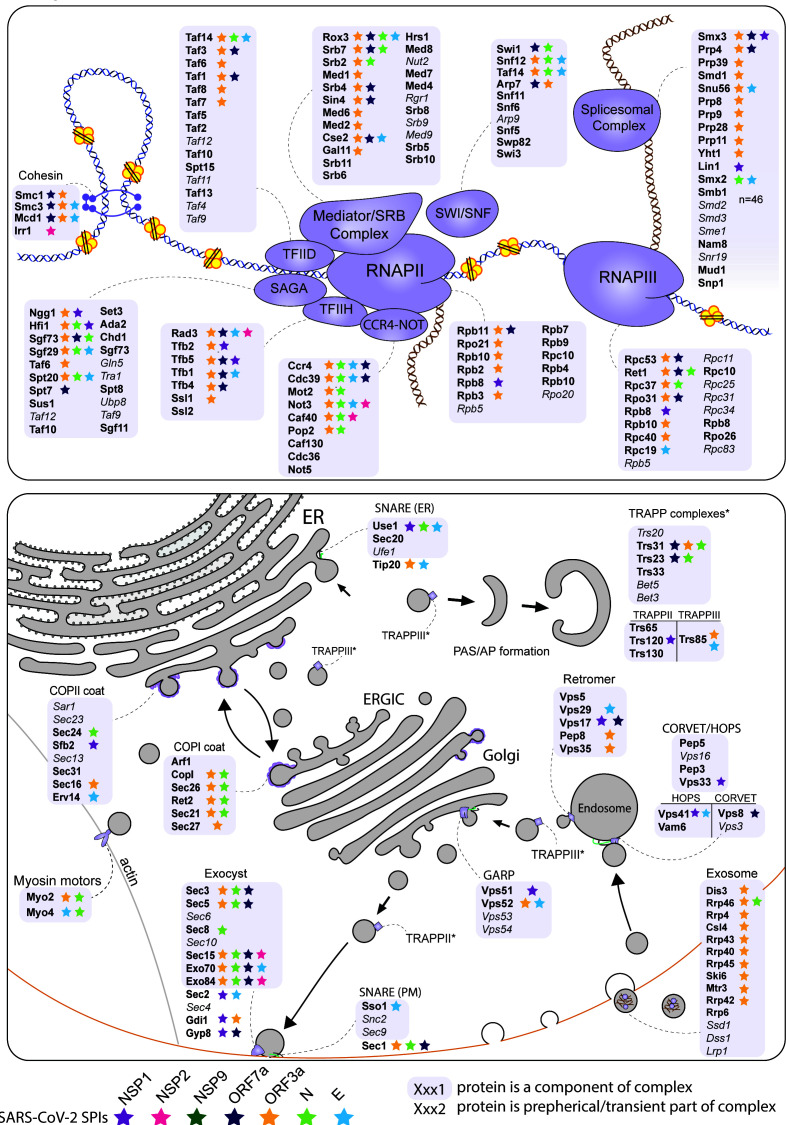
FIGURE 5: Mapping the SARS-CoV-2 yeast SPIs onto the transcription and trafficking pathways in yeast. The SPIs produced by the seven SARS-CoV-2 proteins are mapped onto complexes involved in transcription (top) and trafficking (bottom). The grey boxes encompass complexes and proteins written in grey and italics were not present in our screen. The stars indicate which proteins were SPIs with the colours corresponding to the seven SARS-CoV-2 proteins.

The analysis of enriched processes in the yeast SPIs with SARS-CoV-2 proteins (**Figure 3D**) and the comparison of hoSPIs with genome-wide screens of SARS-CoV-2 in mammalian cells (**Figure 4B** and **4C**), showed that transcription and vesicle trafficking were strongly affected by forced association of SARS-CoV-2 proteins. We next asked which protein complexes involved in these processes were enriched in the yeast SPI data. For vesicle trafficking we found that exocyst, the tethering complex and COP1 vesicle coat were all enriched with SARS-CoV-2 proteins (Data S6). For transcription and RNA metabolism, we identified many complexes that were enriched including the transcription elongator factor complex, mediator complex, SAGA complex and RNA polymerase II transcription factor complex (Data S6). Therefore, we mapped the SARS-CoV-2 SPIs onto the proteins involved in both transcription and vesicle trafficking to highlight this overlap (**Figure 5**). Notably, many components of the ERGIC compartments produced yeast SPIs with SARS-CoV-2 proteins.

### SARS-CoV-2 NSP1 disrupts membrane trafficking at the vacuolar membrane

**Figure 6:**
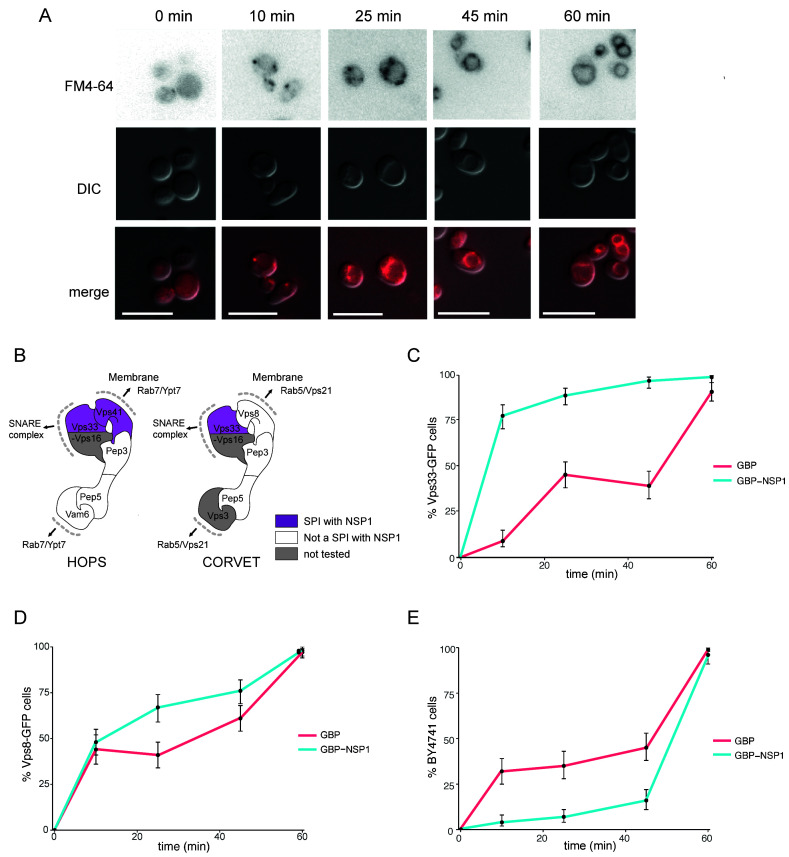
FIGURE 6: Effect of NSP1 SPI at the HOPS complex. **(A)** Uptake of FM4-64 dye to the vacuolar membrane. BY4741 cells were grown in medium containing 4 nM FM4-64 and images taken every 10-20 minutes. The progression of the dye to the vacuolar membrane is clear within 60 minutes. The scale bar is 10 µm. **(B)** HOPS and CORVET complexes are illustrated based on electron microscopy 3D reconstruction [28] and functional domains [68]. Vps33 and Vps41 were identified as SPIs in the NSP1 screen and are highlighted in purple accordingly. **(C)** Following a 90-minute induction of GBP or NSP1-GBP expression in a Vps33-GFP strain, FM4-64 was added, and vacuolar staining of cells was quantified at 10, 25, 45 and 60 minutes from dye addition. Error bars indicate 95% binomial confidence intervals. **(D)** The experiment is as in panel C, but using a Vps8-GFP strain. **(E)** The data is as in panel C, but with a strain that lacks any GFP, BY4741.

The HOPS/CORVET complexes are involved in the tethering and transport of vesicles within the cell and therefore provide good candidate complexes that might be manipulated by a virus. Furthermore, HOPS has been identified as an interactor with SARS-CoV-2 ORF3a [27]. Therefore, we wanted to ask whether the SARS-CoV-2 SPIs with yeast HOPS/CORVET were affecting membrane trafficking. In order to characterise a phenotype of a specific SPI, we devised an endocytosis assay with Biotracker Red FM4-64 dye to investigate SPIs involved in vesicular trafficking. The amphiphilic FM4-64 dye intercalates into yeast cellular membranes and, over ~60 mins, moves through endocytic vesicles to reach the vacuolar membrane (**Figure 6A**), where the HOPS complex facilitates vesicle tethering [28]. The HOPS complex had multiple SPIs with ORF7a, NSP1, and E (**Figure 5**); we focused on the SPIs with NSP1 (**Figure 6B**), since this viral protein is known to be multi-functional but with no known role in vesicle trafficking. NSP1-GBP was transiently expressed using a galactose-inducible promoter in Vps33-GFP, Vps8-GFP and BY4741 (untagged) strains before applying FM4-64 dye and imaging over 60 mins (**Figure 6C-E**). Both GFP-tagged strains exhibited faster vesicle uptake with NSP1-GBP than the GBP only control. Curiously, the presence of NSP1-GBP in the wild-type control had the opposite effect. These data demonstrate that NSP1 is capable of disrupting membrane trafficking in yeast when recruited to HOPS, raising the hypothesis that it may affect HOPS function in mammalian cells.

## DISCUSSION

Yeast has been used as a model system to study fundamental disease processes including ageing, cancer, prion-like diseases and neurodegeneration [29]. Many yeast genes have functional orthologues in metazoans and fundamental processes including cell division, cell death, protein homeostasis, RNA metabolism, metabolism, vesicular transport and signalling pathways are conserved with mammalian cells [30–36]. However, relatively few studies have attempted to examine viral proteins in yeast. Key exceptions to this include the use of yeast as a bioreactor to produce viral antigens, [37] and individual proteins from DNA viruses such as Hepatitis B virus and Epstein Barr virus have been studied in yeast [38, 39]. Studies on Human Immunodeficiency Virus-1 (HIV-1) in yeast have helped to elucidate the functions of several specific viral proteins including the RNA trafficking protein, Rev [40], Vpr [41], and the protease [42]. Additionally, expression of the HIV-1 integrase is lethal in recombination deficient yeast [43], consistent with the function of the integrase during the viral life cycle in mammalian cells.

There have been various studies of positive sense, single strand RNA viruses including Hepatitis C virus, HIV and the SARS family of viruses [44–47], which can replicate in yeast; although we are not aware of attempts to replicate SARS-CoV-2 in yeast. Negative-sense RNA viruses have also been amplified in yeast. An influenza replicon system consisting of viral RNA and viral RNA-dependent RNA polymerase activity was established in yeast. This system was used to identify factors required for viral replication including a homologue of human RNA synthesis stimulatory host factor [48]. As much of a cell's DNA/RNA synthesis and trafficking machinery is conserved between yeast and mammalian cells [49–51], yeast can be a valuable tool for studying viral activity within the cell.

More recently, yeast has been used for genetic engineering of DNA and RNA viruses using transformation-associated recombination (TAR) cloning [52–56] and a recent study has been successful in establishing a synthetic genomics approach to rapidly reconstruct infectious SARS-CoV-2 in yeast [57].

These studies serve two distinct purposes. First, the findings can help to elucidate the function of viral proteins, discussed above and second, if lethal phenotypes can be identified in yeast, then the system provides a tractable tool to identify inhibitors [43]. However, the expression of most individual viral proteins in yeast is not lethal. Consequently, finding phenotypes associated with the expression of viral proteins in yeast is limited.

Here we used the SPI system to identify interactions between specific SARS-CoV-2 proteins and yeast proteins that produce a growth defect (these binary interactions are termed SPIs). For each of these seven SARS-CoV-2 proteins we screened ~4000 interactions and identified 1623 SPIs, with 1171 yeast GFP proteins, of which 849 have human orthologues. Two of the SARS-CoV-2 proteins produced relatively few SPIs (NSP2 and NSP9), and the other five viral proteins produced numerous SPIs (>200) within yeast. These data show that even when expression of viral proteins in yeast does not cause a growth defect, phenotypes can be readily identified by association of the viral protein with specific host proteins. It is possible that the SPI phenotypes are caused simply by a non-specific disruption of the host protein and are not informative of viral protein function. However, the data presented here suggest that the SPIs are biologically relevant. First, we have removed from our data yeast proteins that are frequently identified as SPIs, thus minimising non-specific effects. Second, the yeast SPIs for the SARS-CoV-2 proteins are not all the same but are distinct both from each other and from those produced by other yeast proteins (**Figure 3 B, C**). Third, the viral SPIs are consistent with known functions of both SARS-CoV-2 proteins and proteins from other coronaviruses. Collectively, these data argue that the SPI system in yeast can be used to shed light on viral protein function.

Despite the evolutionary distance, many pathways are conserved from yeast to humans and human orthologues have been identified for many yeast proteins. In addition, some human specific pathways contain proteins with yeast orthologues due to evolutionary repurposing. We identified hoSPIs with several conserved and human specific pathways, which play a role in viral infection. N hoSPIs contained proteins of the 20S proteasome and eIF2B complex involved in antigen receptor-mediated signalling pathway. EIF2B is a known viral target for evasion of translation inhibition of viral RNA in other viruses [58], and a recent study has shown that the 20S proteasome promotes degradation of N in a PA28-dependent manner [59]. Furthermore, N hoSPIs included proteins involved in the assembly of filopodia and, concordantly, growth of filopodia has been identified as a striking phenotypical characteristic of SARS-CoV-2-infected human colon cells [60]. NSP1 hoSPIs included the nonopioid sigma receptor SIGMAR1, which was recently identified as host-dependency factor for SARS-CoV-2 infection and represents a potential drug target for treatment of COVID-19 [61, 62]. The majority of proteins forming the exosome RNAse complex were identified as hoSPIs with ORF3a. Exosomes are involved in host-cell response in COVID-19 patients and were also shown to accumulate viral proteins suggesting that SARS-CoV-2 potentially hijacks the endocytic pathway [63].

The two most striking cellular processes that produced SPIs with the SARS-CoV-2 proteins were in RNA metabolism and vesicle trafficking. The first of these includes numerous components involved in transcriptional regulation, including RNA polymerase II and associated regulatory components (**Figure 5**).

Vesicle trafficking was the second major category of yeast SPIs identified with SARS-CoV-2 proteins. Imaging of SARS-CoV-2 infected epithelial lung cells shows that the Golgi apparatus is fragmented to form viral replication organelles made up of double-membrane vesicles (DMV) [64]. The yeast SARS-CoV-2 SPIs overlap with many proteins located at the endomembrane (**Figure 5**). Additionally, six of the seven SARS-CoV-2 proteins tested produced SPIs with components of the peroxisome, indicating a potential role of viral proteins in perturbing peroxisome function. Notably, a recent study described dramatic changes in structure and composition of peroxisomes in SARS-CoV-2 infected cells [65]. ORF14 was identified as direct interactor to human PEX14, suggesting that peroxisomes are direct targets of SARS-CoV-2 infection.

Multiple studies indicate that ORF3a interacts with hVPS39/Vam6, a subunit of the HOPS complex, blocking SNARE complex assembly and autophagy [27, 66, 67]. NSP1, E and ORF7a all produced SPIs with components of the HOPS/CORVET complex - although none with hVSP39/Vam6. We show that NSP1 influences endocytosis when recruited to Vps33. This SPI phenotype may be mediated through affecting Vam6's ability to interact with the SNARE complex [68].

Some of the SARS-CoV-2 SPIs match interactions identified from studies with other coronaviruses. For example, E protein from SARS-CoV-1 has been shown to bind to human ATP pump subunit ATP1A1 [10]; the yeast orthologue of ATP1A1, Ena2, has a SPI with SARS-CoV-2 E. Consequently, our data show that the SPIs produced by the SARS-CoV-2 proteins in yeast can indicate their sites of function during viral infection in mammalian cells.

We also identified SPIs that suggest novel functions of the viral proteins in eukaryotic cells. For example, we identified SPIs between N and ORF3a with the coatomer complexes and exocyst (**Figure 4C, 5**). These pathways are good candidates for proteins that could mediate viral secretion and merit further investigation. The exocyst complex mediates vesicle-membrane tethering and secretion and may be manipulated to accelerate viral release. The coatomer complex aids movement of vesicles between the Golgi, the ER and the ER-Golgi intermediate compartment (ERGIC). It is known that coronavirus remodels the ERGIC to facilitate viral synthesis [69]. A recent CRISPR screen in human cells infected by SARS-CoV-2 identified subunits of the exocyst as potential host factors required for infection [70].

Since the SPIs indicate slow or no growth when the viral protein is recruited, this system could lend itself well to identifying small molecule inhibitors of the specific functions of viral proteins. Yeast has previously been used to identify both mode of action of small molecules [71] and also identify new drugs for human disease [72]. Additionally, yeast has been used as a tool to screen for drugs that inhibit viral proteins, including coronaviruses [73, 74]. However, these assays typically involve identifying a clear phenotype of a viral protein in yeast. The SPI system provides a tool that can rapidly identify such phenotypes and provide a tractable screening system. Additionally, the SPI assays could be used to screen variants of viral proteins for altered functions. For example, ORF3a mutations are associated altered mortality [75], hence understanding the altered function of these variants is of interest. In summary, this work establishes the SPI system as a powerful model for studying viral protein function in a simple eukaryotic system.

## MATERIALS AND METHODS

### Yeast Methods

All yeast strains used in this study are listed in Table S1. GFP and CFP strains are based upon BY4741 (*his3*Δ*1 leu2*Δ*0 met15*Δ*0 ura3*Δ*0)* [76, 77]. CFP strains were generated from the GFP library by Cas9-assisted tag switching (CATS) [78]. The universal donor strain (UDS) is a derivative of W303 (*can1-100 his3-11,15 leu2-3,112 ura3-1 RAD5*) [79]. Yeast cells were cultured in standard growth medium with 2% carbon source [80]. Plasmids were generated by gap-repair cloning directly in yeast or by using the NEBuilder HiFi assembly kit (New England Biolabs) by combining the linearised plasmid background with gene fragments. All plasmids are listed in Table S2. Fragments were synthesised by GeneArt (Thermo Fisher Scientific, USA) and plasmid constructs were validated using Sanger sequencing (GENEWIZ Brooks Life Science, UK).

### Synthetic Physical Interactions

SPI screens were performed as previously described [8, 81]. Arrays of GFP strains were transformed separately with either control or experimental plasmids. For example, for the NSP1 SPI screens either *pCUP1-NSP1-GBP,* or as controls*, pCUP1-GBP* or *pCUP1-NSP1* were used (*pCUP1* is the promoter from the yeast *CUP1* gene and *GBP* encodes the GFP binding protein). Selective ploidy ablation (SPA) was used to introduce plasmids into arrays of query yeast strains comprising ~4000 members of the GFP collection that represent proteins that are expressed in mitotic cells [21, 77]. Briefly, the SPA method utilises a Universal Donor Strain (UDS, W8164-2B), which contains conditionally-active centromeres, transformed with each of the plasmids. These donor strains were then mated with members of the GFP collection arrayed with four replicates on 1536-colony rectangular agar plates using a pinning robot (ROTOR robot, Singer Instruments, UK). The resulting diploids were put through a series of sequential selection steps to maintain the query strain GFP genome and plasmid, while destabilising and then removing the chromosomes of the UDS by growing the cells in 5-FOA and galactose-containing media. Finally, the plates were scanned using a desktop flatbed scanner (Epson V750 Pro, Seiko Epson Corporation, Japan).

### SPI Data analysis

Colony sizes on SPI screening plates were measured using the colony measurement engine tool for ImageJ [82] and the resulting data analysed using the open-source software ScreenGarden [83], which calculates mean log growth ratios of experimental and control plates for each interaction. Interactions which resulted in an average LGR of more than 0.3 in all previous screens or were SPIs (LGR ≥0.4) in more than 40% of previous screens were highlighted as frequent flyers and excluded from analysis. Additionally, GFP-strains with an average colony size of less than 30% compared to the plate median after forced association of the GBP control were defined as sensitive to recruitment of any protein and were excluded from further analysis. The final dataset includes 3394 unique yeast open reading frames.

### Bioinformatics

Spearman's Rank correlation and comparison to yeast screens from Berry and colleagues were calculated using RStudio. Centroid-linkage clustering of SPIs was performed using the Cluster 3.0 software [84] and visualised using Java TreeView 1.1.6 [85]. Gene Ontology (GO) analysis of SPIs from each screen was performed using the *GOrilla* web application [86] for enrichment of GO terms for biological processes with a two (target and background) list approach. Enrichment was identified with a hypergeometric overrepresentation test, and a p-value cut-off of 10^-3^ was used. The GO ratio of each enrichment is defined as the number of genes from the target list with a GO term/number of genes associated with that GO term. A GO ratio of 1 indicates that all genes with a specific GO term were identified in the screen. Enrichment of process terms with very broad descriptions were excluded.

### Microscopy

We used epifluorescence microscopy to determine the cellular localisation of FP-tagged proteins. Cells were grown to log-phase and mixed with 0.7% low melting point agarose in growth medium on glass microscope slides. A Zeiss Axioimager Z2 microscope (Carl Zeiss AG, Germany) was used to image cells using a 63x 1.4NA apochromatic oil immersion lens. Fluorescence was excited using a Zeiss Colibri LED illumination system (GFP=470 nm, YFP=505 nm, and RFP=590 nm) and differential interference contrast (DIC) prisms were used to enhance the contrast in bright field. The emitted light was captured using a Hamamatsu Flash 4.0 Lte CMOS camera with FL-400 (6.5 µm pixels, binned 2x2). Exposure times were adjusted to ensure that signal intensities remained below saturation and remained identical between control and experimental images. Images were acquired using the Zen software (Zeiss) and analysed and prepared using the Icy BioImage Analysis unit (version 2.0.3.0) [87] and FIJI/imageJ [88].

### FM4-64 Endocytosis assay

Membrane trafficking was measured using the lipophilic styryl dye FM4-64 (N-(3-triethylammoniumpropyl)-4-(6-(4-(diethylamino)phenyl)hexatrienyl) pyridinium dibromide, MilliporeSigma, Germany) [89]. To stain the cells, cultures were grown overnight to log phase in synthetic complete (SC) media with raffinose before inducing expression with media containing galactose for 1.5 hours. Yeast cells were collected by centrifugation, resuspended in SC with glucose and 4nM FM4-64 and incubated at 30°C. Cells were imaged with epifluorescent microscopy 10, 25, 45 and 60 min following dye addition.

## SUPPLEMENTAL MATERIAL

Click here for supplemental data file.

All supplemental data for this article are available online at www.microbialcell.com/researcharticles/2021a-klemm-microbial-cell/.

## Abbreviations:

AP-MS: – affinity purification mass spectrometry;
COVID-19: – coronavirus disease;
E: – envelope;
ERGIC: – ER-Golgi intermediate compartment;
FDR: – false discovery rate;
GBP: – GFP-binding protein;
GFP: – green fluorescent protein;
GO: – gene ontology;
hoSPI: – human orthologues of the SPI;
IP: – immuno-precipitation;
LGR: – log growth ratio;
N: – nucleocapsid;
NSP: – non-structural protein;
ORF: – open reading frame;
RFP: – red fluorescent protein;
SARS-CoV-2: – severe acute respiratory syndrome coronavirus 2;
SPI: – synthetic physical interaction.
