# Supplementary material for: Forced association of SARS-CoV-2 proteins with the yeast proteome perturb vesicle trafficking
Source: Microb Cell. 2021 Oct 27;8(12):280–96. doi: 10.15698/mic2021.12.766 (PMC8642885; doi:10.15698/mic2021.12.766)
Supplement: Supplementary file 1 [file mic-08-280-s01.pdf]

Supplementary Figures and Tables

Figure S1.
SPI plasmids expressing SARS-CoV-2 genes under a constitutively active *CUP1* promoter were transformed into W8164-2B yeast and growth was measured overnight using a microplate reader. Growth curves were calculated using the mean OD_{600} of 10 replicates per strain. The growth rates (right panels) are calculated as the maximum slope of the curve. The top panels indicate the plasmids that were used for 'Gene of Interest' (GOI) controls, i.e. each express an untagged version of the SARS-CoV-2 protein. The lower panels indicate the expression of the SARS-CoV-2 proteins tagged with GBP-RFP. 'CTR' indicates either an empty plasmid (top panel) or a plasmid expressing GBP-RFP not linked to a viral protein (bottom panel). "n.s." indicates not significant, in that differences in growth rate were not statistically different with a Student's *t*-Test. The error bars show standard deviation.

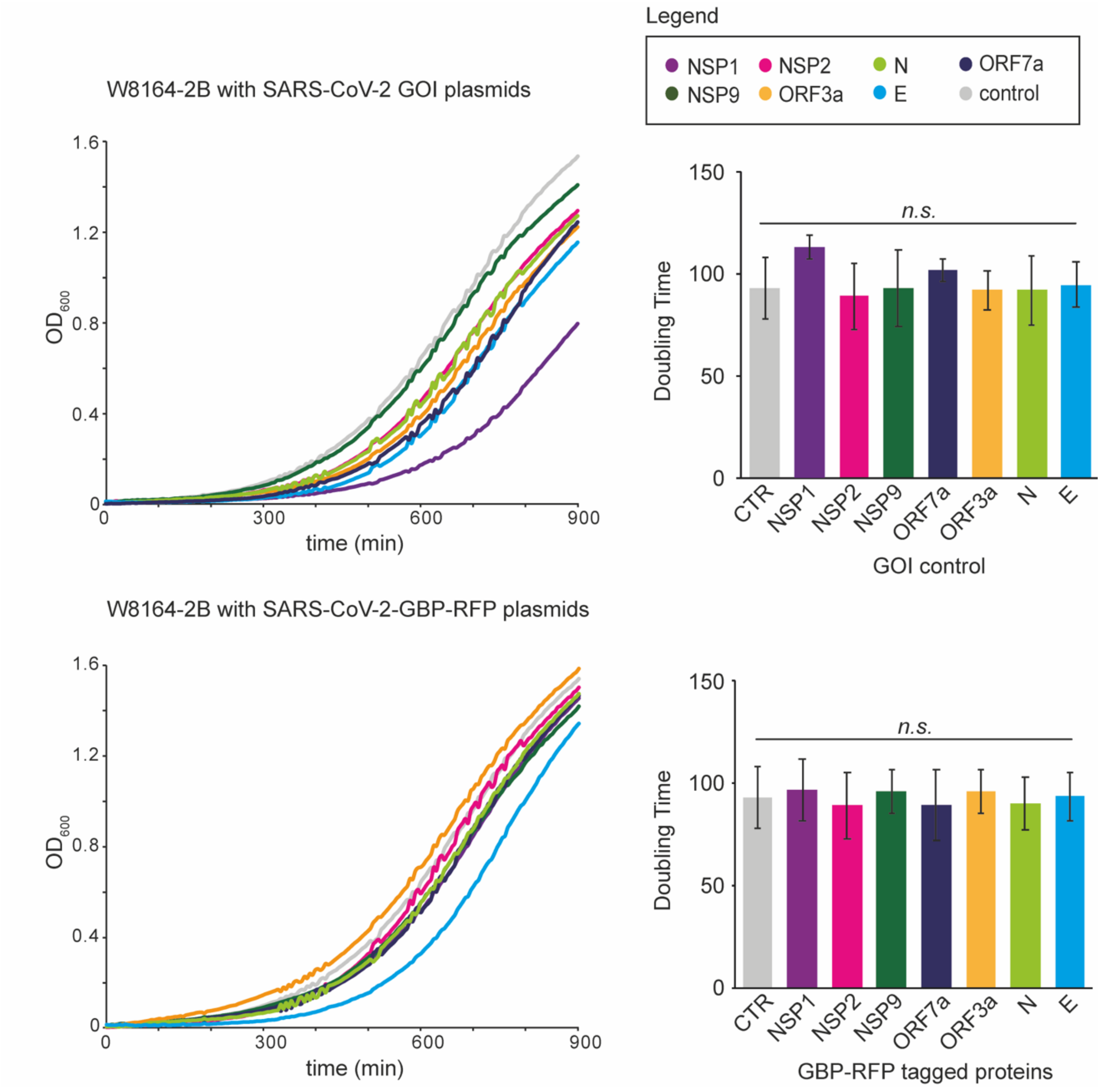

Figure S2.
(**A**) Since GBP-tagged proteins can bind to GFP but not CFP, GFP-tagged yeast strains were transformed into CFP-tagged strains using Cas9-assisted tag switching (CATS) [77]. The illustration shows the G418 selection marker (KAN-MX) and the *HIS3* marker (HIS-MX). (**B**) A set of marker strains for different cellular localisations were validated using fluorescence imaging. CFP-tagged proteins are not detected when exposed to YFP excitation (505 nm), whereas GFP-signals are detectable in both, the CFP (445 nm) and YFP (505 nm) excitation. The blue and yellow channels are shown in greyscale for clarity, the scale bar is 10 μM.

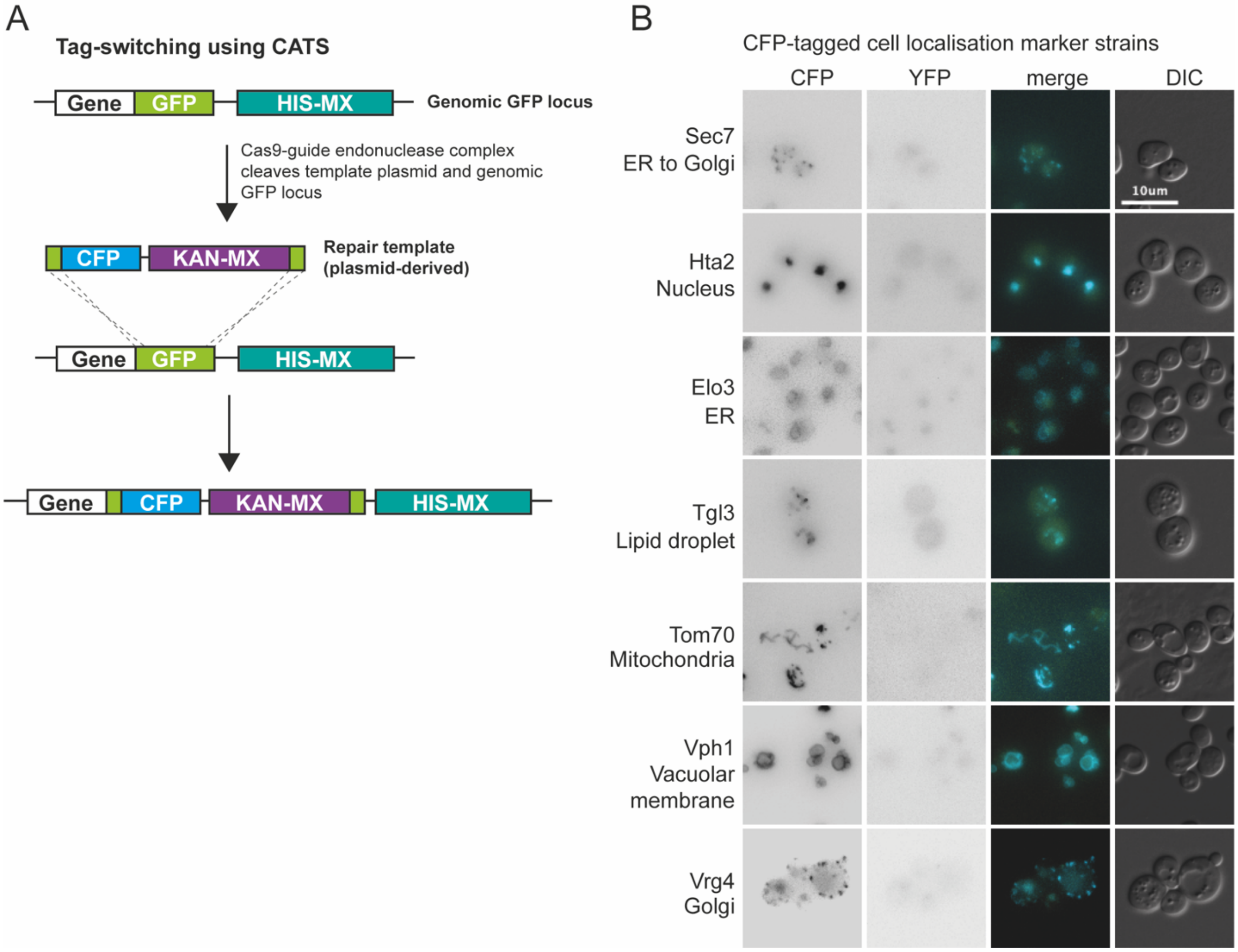

Figure S3.
(**A**) Colocalisation of SARS-CoV2 proteins expressed from SPI plasmids and the GFP-tagged histone Hta1. The green and red channels are shown in greyscale for clarity, the scale bar is 10 µM. (**B**) Colocalisation of SARS-CoV2 proteins expressed from SPI plasmids and the GFP-tagged COPI-vesicle component Sec26. The green and red channels are shown in greyscale for clarity, the scale bar is 10 µM. (**C**) A list of GFP-tagged strains analysed for colocalisation with SARS-Cov2 proteins.

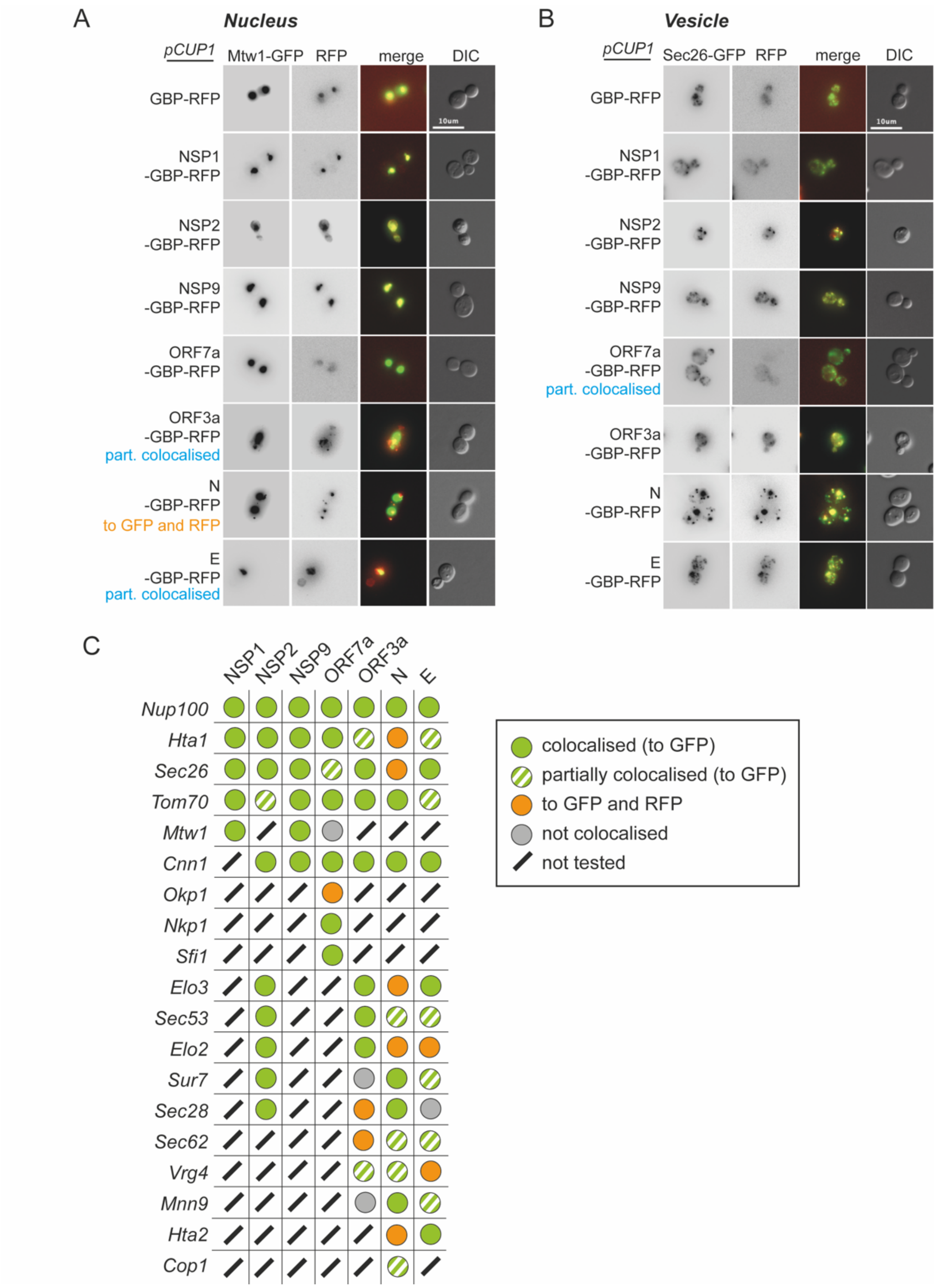

Figure S4.
(**A**) For each SPI screen there were two control plasmids, one expressing the SARS-CoV-2 “gene of interest” (GOI) and one expressing GBP-RFP. In addition, the experimental plasmid encoded the GOI linked to GBP-RFP. (**B-H**). Each screen was compared to both controls. Mean LGRs were calculated based on the average LGR of the two controls. The correlation of data for the two controls is shown for each screen. Dotted lines indicate LGR cut-off values for growth defects (LGR ≥ 0.4). The majority of growth defects are well-correlated between controls.

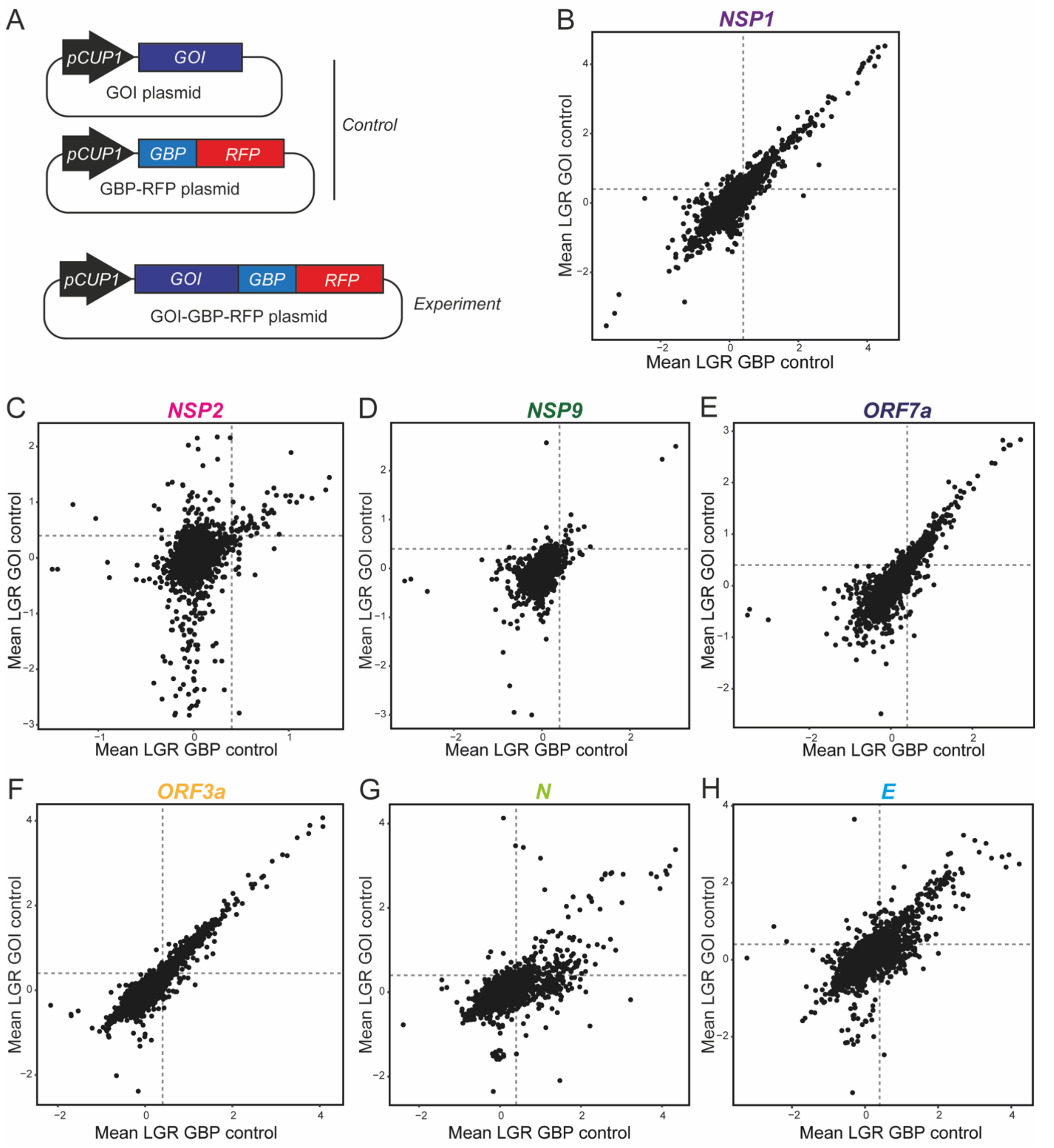

Figure S5:

(**A-D**) LGR of 89 SPIs selected for validation screens (with 16 replicates) with N, Orf7a, Orf3a and E were plotted (y axes) against LGR of the same strains from the proteome-wide SPI screens (x-axes). The dotted lines show the cut-off value of LGR ≥ 0.4 used for both proteome-wide and validation screens. (**E**) The proportion of SPIs that validate for 0.1 intervals of LGRs from the proteome-wide screen ranging from 0.1-0.2 to 0.9-1.0 for the four validation screens combined. The validation rate is around 70-80% for any LGRs between 0.3-0.8 and 90-100% for LGRs between 0.8 and 1. Error bars show the exact binomial 95% confidence intervals. (**F**) The proportion of strains that validate using different cut-off values in the proteome-wide screen. Note that for lower cut-off values, the validation rate remains high since most validated strains were SPIs in the proteome-wide screen. Error bars show the exact binomial 95% confidence intervals. (**G**) Examples of raw data taken from the proteome-wide screens (arrayed with 4x replicates, left) and validation screen (arrayed with 16x replicates, right).

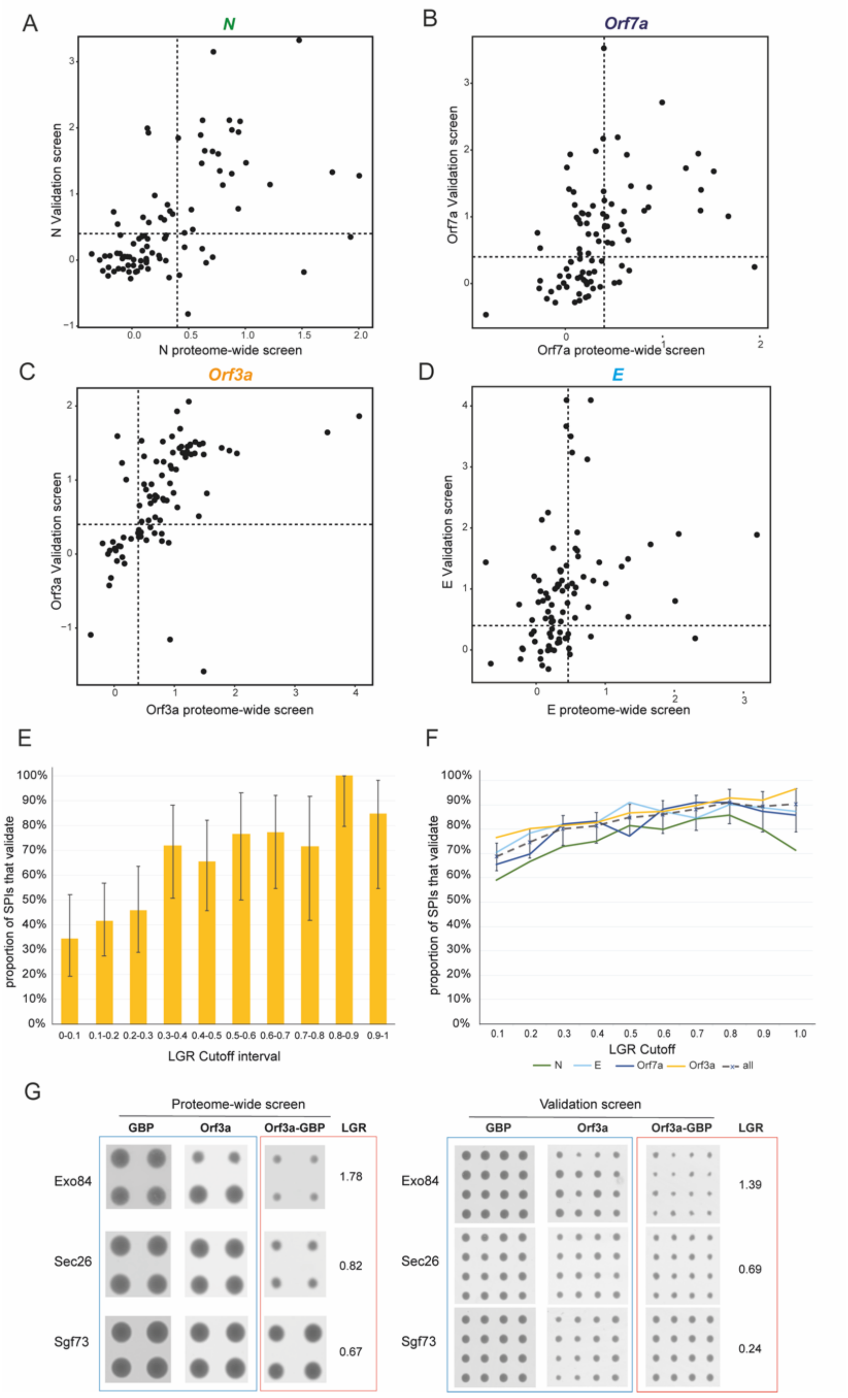

Figure S6:
(**A**) The Venn diagram shows the overlap of the hoSPIs with the two CRISPR loss of function screens performed in mammalian cells. The 15 proteins that are common to all three screens are listed. (**B**) The Venn diagram shows the same data as panel A, but with the inclusion of the mass-spectroscopy data from Gordon et al. The overlapping proteins between the hoSPIs and the mass-spectroscopy data are listed.

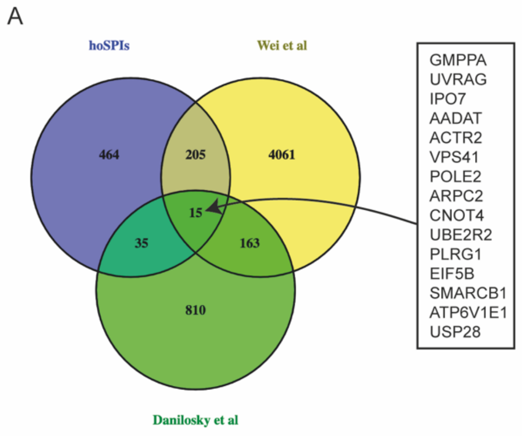

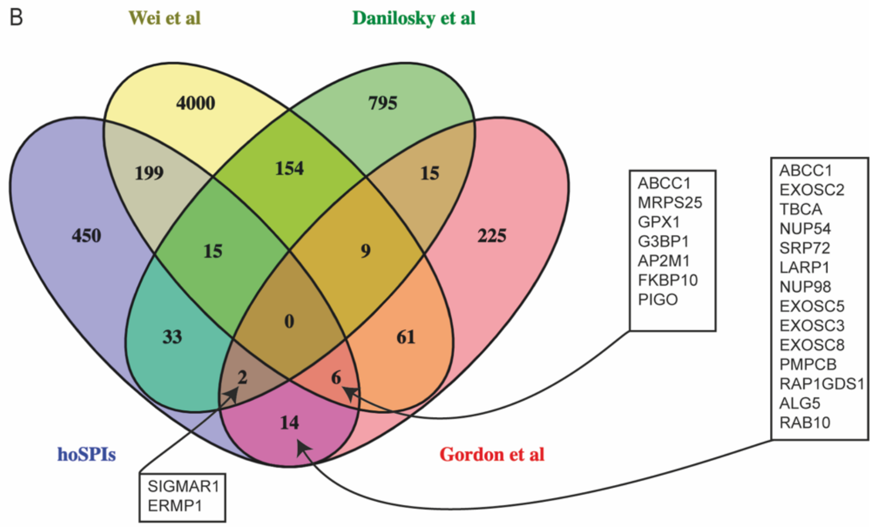

Table S1: List of yeast strains used in this study

**Strain**	**Genotype**	**Source**
W8164-2B/UDS	*MATα CEN1-16::Gal-KI-URA3*	Reid et al. 2011
GFP library/ BY4741-derived	*MAT*a *his3Δ1 leu2Δ0 met15Δ0 ura3Δ0 XXX-GFP:HIS3*	Huh et al. 2003
CFP library/ BY4741-derived	*MAT*a *his3Δ1 leu2Δ0 met15Δ0 ura3Δ0 XXX-CFP:HIS3::KAN*	This study

Table S2: List of plasmids used in this study

**Plasmid**	**Genotype (all contain ARS209, CEN6 and AMP)**	**Source**
pWJ1512	*pCUP1 LEU2*	Reid et al. 2011
pHT4	*pCUP1 GBP-RFP LEU2*	Olafsson and Thorpe 2015
pHT807	*pCUP1 NSP1 LEU2*	This study
pHT808	*pCUP1 NSP1-GBP-RFP LEU2*	This study
pHT809	*pCUP1 NSP2 LEU2*	This study
pHT810	*pCUP1 NSP2-GBP-RFP LEU2*	This study
pHT811	*pCUP1 NSP9 LEU2*	This study
pHT812	*pCUP1 NSP9-GBP-RFP LEU2*	This study
pHT813	*pCUP1 ORF7a LEU2*	This study
pHT814	*pCUP1 ORF7a-GBP-RFP LEU2*	This study
pHT821	*pCUP1 N LEU2*	This study
pHT822	*pCUP1 N-GBP-RFP LEU2*	This study
pHT823	*pCUP1 ORF3a LEU2*	This study
pHT824	*pCUP1 ORF3a-GBP-RFP LEU2*	This study
pHT825	*pCUP1 E LEU2*	This study
pHT826	*pCUP1 E-GBP-RFP LEU2*	This study
pHT199	*pGAL1 GBP-RFP LEU2*	Olafsson and Thorpe 2016
pHT842	*pGAL1 NSP1-GBP-RFP LEU2*	This study
